# Thinking about possibilities: mechanisms, ontogeny, functions and phylogeny

**DOI:** 10.1098/rstb.2021.0333

**Published:** 2022-12-19

**Authors:** Jonathan Redshaw, Patricia A. Ganea

**Affiliations:** ^1^ School of Psychology, The University of Queensland, Brisbane 4072, Australia; ^2^ Department of Applied Psychology and Human Development, University of Toronto, Toronto, Canada M5S 1V6

**Keywords:** possibility, modal logic, mental simulation, counterfactual reasoning, causal reasoning, decision-making

## Abstract

Humans possess the remarkable capacity to imagine possible worlds and to demarcate possibilities and impossibilities in reasoning. We can think about what might happen in the future and consider what the present would look like had the past turned out differently. We reason about cause and effect, weigh up alternative courses of action and regret our mistakes. In this theme issue, leading experts from across the life sciences provide ground-breaking insights into the proximate questions of how thinking about possibilities works and develops, and the ultimate questions of its adaptive functions and evolutionary history. Together, the contributions delineate neurophysiological, cognitive and social mechanisms involved in mentally simulating possible states of reality; and point to conceptual changes in the understanding of singular and multiple possibilities during human development. The contributions also demonstrate how thinking about possibilities can augment learning, decision-making and judgement, and highlight aspects of the capacity that appear to be shared with non-human animals and aspects that may be uniquely human. Throughout the issue, it becomes clear that many developmental milestones achieved during childhood, and many of the most significant evolutionary and cultural triumphs of the human species, can only be understood with reference to increasingly complex reasoning about possibilities.

This article is part of the theme issue ‘Thinking about possibilities: mechanisms, ontogeny, functions and phylogeny’.

## 1. Introduction

Notions of *possibility* have a long and storied history in fields as diverse as philosophy, literature and physics. In classical Greece, Aristotle [1] contemplated the human proclivity to conceive of events as not necessarily true or false, but rather contingent or variable, anticipating formal systems of modal logic developed over two millennia later [2–4]. During the Enlightenment, Gottfried Leibniz [5] sought to explain the existence of evil by arguing that God must have chosen to create the ‘best of all possible worlds’, although many other thinkers were not so convinced (e.g. [6–8]). Reflections on possibilities were central to the works of twentieth-century author Jorge Luis Borges [9], whose short stories described, for instance, a vast library containing all possible books of a certain length with unique combinations of letters (in *The Library of Babel*), and a world where every possible event is realized in some alternative timeline (in *The Garden of Forking Paths*). The twentieth century also saw the birth of quantum mechanics, sparking incessant theorizing about whether a single particle can simultaneously occupy multiple possible states; and whether, just as Borges fictionalized, the universe truly forks into every possible path at every quantum juncture [10].

But for all the grandeur of these arguments and ideas, it is clear that thinking about possibilities is also a basic human capacity, predictably acquired by every typically developing child and intimately familiar to adults. We can all draw lines between possible and impossible events, actions and states of affairs, even if we disagree on exactly where those lines should be drawn. We can all form theories about how an effect might have been produced, even if we were not there to witness the actual cause ourselves. And we can all imagine and compare a multitude of possible ways the future might turn out, or speculate about once possible ways the past might have turned out but did not.

Psychological approaches to possibility have taken varied and fragmented forms in recent years, with parallel literatures exploring conceptually overlapping questions but rarely making substantive contact with each other. Some lines of research, for instance, suggest that even human infants [11,12] and rodents [13] can mentally represent mutually exclusive possibilities, whereas other research suggests that preschool-aged children [14–16] and non-human great apes [17] struggle to reason about how such possibilities relate. Some theoretical approaches seek to develop models of possibility representations using concepts borrowed from formal logic and linguistics [18–21], whereas other approaches emphasize temporal reasoning about counterfactual past and potential future episodes [22–25]. It is perhaps unsurprising that scientists from distinct research traditions have explored this topic using the conventional experimental and conceptual tools at their disposal. But how can we reconcile the seemingly contradictory findings from different populations, and how can we consolidate the models that have been formulated in seemingly incongruent terms?

In this compilation, experts from psychology, neuroscience, behavioural economics, biology, linguistics and archaeology offer pioneering empirical and theoretical insights into the cognitive science of possibility. Following other recent efforts (e.g. [26–28]) to explain psychological traits according to Tinbergen's four questions [29,30], the papers are categorized into those broadly focused on the proximate questions of *mechanisms* and *ontogeny*, and those broadly focused on the ultimate questions of *functions* and *phylogeny*. These categories are complementary rather than mutually exclusive, however, and many contributions bear on both proximate and ultimate questions. Indeed, thinking about possibilities takes distinct forms with distinct functions, and the nature of these forms and functions may vary over human development and across the animal kingdom. Although the papers examine different aspects of thinking about possibilities and come from different theoretical perspectives, together they lay the foundation for a new and unified understanding of the capacity.

## 2. Proximate questions: mechanisms and ontogeny

When asking how a human or other animal can imagine and reflect on possibilities, we might consider the question from different levels of analysis. We might, for example, consider the neurophysiological mechanisms supporting the representation of non-actual states of affairs, in terms of either single cell activations, neural oscillations within particular brain areas or activity across entire networks. We might also consider the multifaceted cognitive capacities involved in reasoning about possibilities, especially those that support the mental representation and integration of events removed from the here-and-now. And we might consider the broader social and cultural inputs that could at the very least facilitate, and perhaps even crucially enable, the emergence of sophisticated reasoning about possibilities in human children. Across this theme issue, many of the papers offer novel insights from one or more of these levels of analysis.

### (a) The neurophysiological basis of thinking about possibilities across species

The hippocampus has long been identified as one of the central brain nodes supporting the mental construction of visuospatial scenarios in both humans [31,32] and non-humans [33]. Over the past two decades, for instance, an accumulation of evidence suggests that hippocampal place cells in rodents support the ‘replay' and ‘preplay' of past and potential future movements through space (e.g. [34,35]). In this issue, Comrie *et al*. [36] propose that the hippocampus has an even more general function: to support the mental exploration of alternative possibilities in space and time. For example, the authors point to recent findings [13] indicating that theta oscillations in the rodent hippocampus can maintain simultaneous representations of paths to the left and the right of a fork in a maze (or in front of and behind the animal). Broadly, such findings suggest that even relatively simple mammalian brains can support at least basic dual representations of alternative possibilities in the immediate environment.

But the hippocampus is not the only brain region crucial to the generation of possible scenarios, and elsewhere in this issue, an fMRI study by Khoudary *et al*. [37] highlights distinctive roles for specific regions. The authors asked human participants to imagine how negative past personal experiences could have turned out better, either by envisioning that they had taken an alternative course of action or by envisioning that the contextual circumstances surrounding the event had been otherwise. Results showed that areas within the brain's ‘default mode network’ (DMN)—which plays a predominant role in the construction and maintenance of imagined scenarios [38,39]—were active during both forms of counterfactual thinking. Distinct regions beyond the DMN, however, were differentially activated depending on the type of counterfactual imagined. Overall, this finding suggests that the human brain does not support an encapsulated, one-size-fits-all approach to imagining possibilities, and instead this capacity draws on a range of interlinked and differentially engaged neurocognitive components [40].

Questions remain over whether the basic hippocampal mechanisms identified by Comrie *et al*. [36] in rodents are separable from or merely a constituent of the global networks identified by Khoudary *et al*. [37] in humans. After all, rodent replay events, preplay events and theta oscillations correspond to simple navigational routes, whereas human representations of possible scenarios can involve complex interplays between various actions and actors, taking place in roughly real time [41]. Critically, Comrie *et al*. [36] highlight clinical evidence suggesting that the human hippocampus may not be strictly required for mental imagery [42], and emphasize that hippocampally supported representations of possibilities across species ‘can be understood at the level of the brain and need not entail conscious awareness or mental imagery’. As in the theory of mind literature [43–45], therefore, it may be useful to distinguish between *implicit* representations of possibilities on one hand, and *explicit* representations on the other. Under this scheme, an animal that can maintain dual representations of possibilities via hippocampal activity need not be consciously aware of these possibilities at all, let alone understand how two mutually exclusive possibilities relate to one another (also see [24]). As summarized further on (see section ‘Thinking about possibilities in non-human animals’), behavioural studies have only recently begun to make progress on the question of whether non-human animals can reason about possibility relations.

### (b) Early and late developments in human thinking about possibilities

In recent years, multiple lines of research have begun to map the early origins of representing and reasoning about possibilities (and probabilities) in humans, often using behavioural and physiological measures that enable the testing of non-verbal infants [46]. In this issue, Cesana-Arlotti *et al*. [47] build on their previous work [11,12] and present novel pupillometric evidence that even 14-month-old infants can simultaneously represent two mutually exclusive possibilities, which contrasts with previous studies showing that children younger than 4 years struggle to prepare for such possibilities (e.g. [Bibr RSTB20210333C15][17]). The authors attempt to reconcile the discrepancy by suggesting that young children may be limited in their action planning and decision-making abilities, rather than limited in their conceptual understanding of possibilities *per se*. Alternatively, however, one might hypothesize that infants (and children up to a certain age) merely have implicit access to dual possibilities—perhaps drawing on similar neurocognitive mechanisms to those outlined by Comrie *et al*. [36]—without necessarily being able to explicitly envision such possibilities and reason about logical relations between them[Bibr RSTB20210333C24]. Much further work is required to uncover the precise reasons for the apparently contradictory patterns of findings from infants and older children [19].

Explicit reasoning about possibilities can take various forms, as demonstrated by the protracted development of the human imagination. In this issue, Harris [48] reviews evidence suggesting that, by 2–3 years of age, children can imagine and draw appropriate inferences about possible events and situations. Around this age, children can sustain a pretend play situation with a partner, attending to the actions of the partner within the constraints of a shared alternative version of reality [49]; they can imagine a potential location of a missing object and deduce its location upon receiving relevant information [50,51]; and they can deploy linguistic indicators of uncertainty (such as *maybe* and *might*) in situations where they suspect a state of affairs to be true but lack definitive evidence [52,53]. Harris concludes that, while children of this age may typically be restricted to imagining *single* possibilities in situations of uncertainty, they seem to know that such possibilities are not necessarily the case.

Harris' [48] framework provides a useful lens for interpreting some of the new empirical data presented in this issue. In one contribution, Wente *et al*. [54] examine the relationship between pretend play and reasoning about possibilities in 3- to 4-year-old children from Peru and the USA. The counterfactual task in this study required children to mentally simulate what would happen if one aspect of a previously shown causal scenario were altered, such that the children had to generate a single novel possibility to pass. The authors found evidence of correlations between pretend play and performance in the counterfactual task, and—accounting for the variation associated with socioeconomic status [55]—the developmental trajectory of children's performance roughly matched the developmental trajectory specified in Harris' framework. Also mapping onto Harris' framework is Goulding *et al*.'s [56] study of 3-year-old children's ability to identify their own past and future preferences. The results show that many children of this age, for instance, can accurately simulate that they would have preferred to have toy blocks as a baby but will prefer to have a car as an adult.

But although young children appear able to explicitly envision single possibilities, and may even recognize the uncertainty surrounding such possibilities, Grigoroglou & Ganea [57] point to evidence suggesting that it takes further time before children fully comprehend the logical relation between two dependent possibilities (A or B). Assuming there is in fact something unique about older children's understanding of possibilities, then a corollary question is just *how* they acquire such understanding. Perhaps it is simply a process of routine biological maturation, such that any child who receives a basic degree of physical and social care develops the capacity as a matter of course. Another intriguing idea, however, is that environmental inputs play a more active role in the emergence of children's grasp of possibilities. In their review, Grigoroglou & Ganea [57] propose an important role for language, such that children observe and use the language of possibility before they fully understand the denoted concepts, and, in turn, their familiarity with this language gradually enables their conceptual understanding to mature. One potential mechanism of this language-concept feedback loop is *polysemy*, or the fact that words used to denote modal concepts tend to have alternative, simpler meanings. For example, children may learn the simple meaning of modals (e.g. *You may go now*) through observation (such as noticing the absence of a physical barrier in the physical world) and then extend these meanings to more abstract, epistemic cases (e.g. *He may be at work*). This type of ‘conceptual bootstrapping’ through linguistic mapping [58] has been proposed for other domains of knowledge (i.e. number knowledge).

The discrepancy between notions of single possibilities and modal possibilities is also a recurring point of contention in the developmental literature on counterfactual reasoning. One research tradition takes a broad definition of counterfactual reasoning, including any forms of thinking involving the generation of single alternatives to reality [59,60]—as in Wente *et al*.'s [54] study. Another research tradition, by contrast, includes only the specific form of thinking where one imagines what the present situation would be like had a past event turned out differently [61,62]. This form of counterfactual reasoning ostensibly relies on the recognition that, although past events are currently fixed, they once had multiple possible outcomes [24,25,63]. Such reasoning is thought to be quite late developing, with various lines of evidence suggesting that children do not acquire it until around 4–6 years of age [64–66]. Similarly, whereas Goulding *et al*.'s [56] study suggests that many 3-year-olds can readily generate a single future possibility, only by 4–6 years of age do most children show consistent evidence of preparing for two alternative future possibilities [14,16,67].

In summary, although it remains unclear just how the mechanisms supporting the basic representation of possibilities in non-human mammals and infants relate to the mechanisms supporting thinking about possibilities in older children and adults, it is clear that the expression of such thinking in behaviour, at least, undergoes a protracted development in humans. A key question for future research will be to uncover whether this gradual development merely reflects improvements in action planning and decision-making, as Cesana-Arlotti *et al*. [47] would have it, or whether it reflects genuine conceptual transformation, as Harris [48] and Grigoroglou & Ganea [57] would have it.

### (c) Cultural influences on human thinking about possibilities

Sophisticated thinking about possibilities may not only be transformed by linguistic input, as Grigoroglou & Ganea [57] suggest, but also by cultural forces more broadly. Cultures of course vary in the events that people typically consider to be possible versus impossible, especially in regard to existential questions such as the origin of the universe and life, or the potential for supernatural entities and causation [68]. From a more practical standpoint, however, Vale *et al*. [69] highlight how human cultural activities that readily meet ongoing needs, such as food and shelter, can free up time and resources to enable individuals to reflect on novel future possibilities that can then become goals worth pursuing. Furthermore, cultural time-keeping methods such as oral or physical calendars can allow us to mentally generate and communicate about possibilities that might take place at precise and agreed upon moments in the future. On the whole, it seems undeniable that many human cultural practices enhance the quantity and quality of the possibilities that individuals and groups envision.

Other cultural influences, however, may curtail and bias the generation of possibilities. In a series of studies, Epstude *et al*. [70] provide evidence that political polarization leads to a divergence in the type of counterfactual past events that people tend to entertain and endorse. The authors found that, among USA residents, highly partisan Democrats and Republicans were more likely than non-partisans to judge counterfactuals as ‘plausible’ if those counterfactuals aligned with their political views (e.g. when asked to reflect on what would have happened if Trump or Biden had not passed some contentious tax cuts). The findings also showed that partisans were more likely than non-partisans to blame a leader from the opposition party for a negative past event that ‘nearly’ occurred but did not (e.g. the USA and North Korea going to war during Trump's presidential term).

Across the contributions to this theme issue, it becomes clear that much of human thinking about possibilities follows a reliable course during development, supported by basic neural mechanisms shared among mammals as well as by mechanisms that are highly differentiated in our species. It is also clear, however, that such thinking varies between and within cultures, with linguistic, socioeconomic and political factors shaping the ways in which children and adults generate and reason about possibilities.

## 3. Ultimate questions: functions and phylogeny

The capacity to think about possibilities takes numerous forms with general and specific developmental trajectories, and also has numerous functions that cut across cognitive and behavioural domains. As the contributions to this issue show, thinking about possibilities can help us learn, choose, reason and judge. And while some forms and functions of human thinking about possibilities are likely shared with non-human animals, others may be unique to our species.

### (a) Thinking about possibilities as a means of learning

Thinking about possibilities enables prospective forms of learning, in that one can imagine potential future actions and consequences without necessarily having to observe these consequences play out in the real world [71,72]. It also enables retrospective learning about counterfactual actions and consequences, as when we imagine what might have happened had we pursued an alternative course of action in the past [73,74]. Such vicarious learning can be especially potent when the imagined consequences are emotionally charged [75], as in the experience of *regret* when comparing a sub-optimal past choice to a superior but counterfactual past choice [76]. Here, Gautam *et al*. [77] demonstrate that even young children experience regret following such counterfactual thinking. Four- to 9-year-old children were shown that a reward they did not obtain was better than a reward they had obtained, and were then asked to report their change in emotion. Results showed that children were more likely to report a negative change in emotion (i.e. regret) if they previously had an opportunity to receive the better reward by making a counterfactual choice. Ultimately, counterfactual emotions like regret may assist children and adults to make better choices when confronted with similar situations in the future [78,79].

As FitzGibbon & Murayama [80] demonstrate, however, learning about counterfactual past possibilities need not be an exclusively mental exercise. The authors discuss recent findings suggesting that both children and adults show ‘counterfactual curiosity’ about alternative outcomes after making decisions, such that they will actively seek out information about such outcomes when the opportunity to attain them has already passed. Puzzlingly, people will seek such information even when it predictably leads to a negative emotional experience, and even when it offers no opportunity for adaptive learning. In one recent study, for example, 4- and 5-year-old children played several rounds of a card selection game, and at the end of each round they would often peek at better, unselected cards, even when they had no chance to replay the round [81]. FitzGibbon & Murayama explain such behaviour by arguing that counterfactual information generally *does* have adaptive learning value, and thus the proclivity to seek it may be biologically hard-wired rather than always being the product of reasoned decision-making. This account would also explain why non-human animals such as rhesus macaques, which may lack a capacity for genuine counterfactual reasoning, nonetheless show curiosity about counterfactual information much like humans [82].

### (b) Thinking about possibilities in decision-making and judgement

In adults, thinking about future possibilities is often measured with intertemporal decision-making tasks, which require participants to make monetary choices between a smaller amount now and a larger amount later (e.g. *would you rather have $15 now or $20 in a month?*). These choices are commonly assumed to index self-control, such that any decision to select the smaller, sooner amount reflects a failure to regulate immediate impulses [83]. As Bulley *et al*. [84] demonstrate, however, this assumption may be unfounded. Across two experiments, the authors gave participants intertemporal choices and then asked them to rate their confidence in having made the right decisions. Contradicting the self-control account, confidence did not vary as a function of whether a participant opted to wait for the delayed reward. Rather, confidence was associated with the difference in subjective valuation between the two possibilities, such that a participant might in fact be highly confident in selecting $15 now if they independently valued that reward-time combination much more highly than $20 in a month. These findings align with recent research [85] questioning self-control accounts of the classic ‘marshmallow test’ for children [86], further showing that comparisons of mutually exclusive possibilities are integral to human intertemporal decision-making.

Thinking about possibilities may also be essential to causal reasoning. Theories have previously identified that counterfactual simulations in particular may be fundamental to human judgements of causality [87,88], such that people tend to judge that *A* caused *B* if and only if *B* would not have eventuated in the absence of *A*. As Gerstenberg [89] points out, however, an alternative possibility is that causal judgements depend on hypothetical simulations of the future. That is, people may judge that *A* caused *B* by imagining a potential future scenario in which *A* is absent and *B* therefore fails to eventuate. Building on previous work [90], Gerstenberg sought to disentangle these explanations by presenting adult participants with scenarios in which counterfactual past simulations and hypothetical future simulations should have in principle produced opposite causal judgements. Results showed that participants' judgements closely matched the counterfactual simulation account, suggesting that people may in fact mentally travel back in time and consider alternative pasts when attributing causality. Intriguingly, although the participants in this study were adults, Gerstenberg concludes that if children of a certain age were to show the same pattern of causal judgements in an equivalent task, then it would suffice to attribute these children with a capacity for counterfactual reasoning. Nonetheless, it is worth noting that, in other contexts, children's causal attributions and counterfactual judgements are often incompatible [91]. The relationship between counterfactual and causal reasoning—and the question of whether one form of reasoning has primacy in human development—will remain subject to debate and further research [92].

Representations of possibilities may not only constrain descriptive judgements, such as those involved in causal attributions, but also prescriptive judgements, such as those involved in moral attributions. In this issue, Acierno *et al*. [93] asked adult participants to make fast or slow judgements about whether a series of actions (e.g. serving a cake containing nuts to a child with a nut allergy, or ‘zapping’ the nuts out of the cake) were morally permissible, and about whether those same actions were possible. Results showed that participants were more likely to judge that (pre-defined) improbable, irrational and impossible actions were immoral when they had to respond quickly than when they had time to deliberate. Furthermore, the correlation between permissibility and possibility judgements was stronger when participants were making fast permissibility judgements than when they were making slow permissibility judgements. The authors explain these findings by suggesting that moral intuitions are constrained by possibility intuitions, such that actions that initially seem to be impossible (or highly improbable) are automatically assumed to be immoral. Acierno *et al*.'s [93] findings provide further evidence that notions of possibility and morality are tightly linked in thought and action [94].

### (c) Thinking about possibilities in non-human animals

On the whole, thinking about possibilities clearly has important functions in human behaviour, highlighting just why natural selection may have favoured the evolution of sophisticated possibility representations in our species. Given the wide range of functions, however, it is also reasonable to suppose that natural selection may more generally favour the emergence of at least low-level, implicit possibility representations across the animal kingdom. Indeed, the neuroscientific data discussed by Comrie *et al*. [36] are difficult to reconcile with any suggestion that non-human animals entirely lack mental representations of possible states of reality. Nonetheless, it remains a point of contention just how sophisticated such representations are in non-human animals [24], just how much explicit insight animals have into these representations [95], and just what functions these representations support across species.

Perhaps the most well-established literature relevant to non-human animals' thinking about possibilities comes from intertemporal decision-making tasks. Whereas human participants are typically asked to make monetary decisions in such tasks, as in Bulley *et al*.'s [84] experiments, animals are often presented with decisions between smaller, immediate food rewards and larger, later food rewards. In humans, a preference for later rewards has been linked to general intelligence [96], and researchers have recently documented similar links in chimpanzees [97] and cuttlefish [98]. Here, Schnell *et al*. [99] extend this line of research in Eurasian jays (*Garrulus glandarius*), a bird from the Corvidae family that is notable for an impressive range of intelligent behaviours [100]. The authors found that the individual jays that tended to wait for larger possible food rewards also tended to perform better on a battery of cognitive tasks assessing memory, spatial knowledge and learning.

Schnell *et al*. [99] interpret their findings from a self-control perspective, arguing that the proclivity to resist immediate temptation may be a fundamental marker of general intelligence across species. This perspective contrasts with Bulley *et al*.'s [84] interpretation of the human data, which views intertemporal decision-making as reflecting an ability to explicitly compare subjective valuations of mutually exclusive possibilities. These alternative interpretations could be reconciled in at least two ways. On one hand, it could be that general intelligence across species is not associated with self-control *per se*, but rather with an individual's proclivity to subjectively value larger, later possible rewards more than smaller, sooner possible rewards. On the other hand, it could be that self-control is indeed associated with general intelligence in non-human animals, as Schnell *et al*. [99] suggest, whereas in humans the relationship is more complex and moderated by metacognition [101,102]. Humans may, for instance, uniquely understand that future rewards are inherently uncertain, and that therefore sometimes the best decision is to take the smaller reward while it is available [103]. In other words, although animal performance may straightforwardly reflect an ability to resist the temptation offered by the immediate reward, humans may also routinely reason about the mutually exclusive possibilities of (i) eventually receiving the larger, later reward, and (ii) not receiving that reward at all.

While not directly addressed in this issue, studies have recently begun to examine whether non-human animals can similarly reason over mutually exclusive possibilities. Interpretations remain contentious [19,24,104,105], but the available evidence suggests that animals (including non-human primates) tend to perform better on ‘epistemic uncertainty’ tasks requiring them to consider two possible locations of a previously hidden reward [106–109] than on ‘physical uncertainty’ tasks requiring them to anticipate two possible future trajectories of a currently visible reward [17,105,110,111]. There are at least four plausible explanations for these conflicting findings. First, in line with Cesana-Arlotti *et al*.'s [47] interpretation of the infant data, non-human animals may possess the conceptual apparatus necessary to solve both types of tasks yet fail the physical uncertainty tasks because of difficulties with action planning and decision-making [105]. Second, animals may be able to reason over mutually exclusive possibilities in situations of epistemic but not physical uncertainty [106]—opposite to the pattern observed in young children [67]. Third, animals may pass the epistemic uncertainty tasks via implicit representations of mutually exclusive possibilities, such as those identified by Comrie *et al*. [36], yet fail the physical uncertainty tasks because they cannot explicitly reason about multiple possible future locations of a currently visible object [24]. Fourth, animals may pass the epistemic uncertainty tasks via some other low-level process such as associative learning, but fail the physical uncertainty tasks because they simulate a single possibility and treat that simulation as reality [19]. A pressing challenge for the field is to identify which of these alternative explanations, if any, is most accurate.

### (d) The evolution of uniquely human thinking about possibilities

Whatever the underlying capacities of non-human animals, it is undeniable that at least some behavioural expressions of thinking about possibilities are confined to the human lineage. In this issue, Langley and Suddendorf [112] review archaeological evidence suggesting that human ancestors were long entertaining and preparing for more complex future possibilities than non-human primates. By around 1.8 Ma, for instance, *Homo erectus* individuals were manufacturing stone tools and transporting raw materials across vast distances [113,114], presumably because they recognized the possibility of using these tools and raw materials in the future. Later, around 500 000 years ago, *Homo heidelbergensis* individuals began assembling compound tools and apparently coordinating their actions in the service of shared goals—activities likely underpinned by an ability to reason through several interconnected steps in a possible causal chain. Only by around 100 000 years ago, however, is there compelling evidence of *Homo sapiens* and Neanderthals burying their dead, potentially reflecting sophisticated thoughts about the abstract possibility of an afterlife.

On the whole, Langley & Suddendorf's [112] analysis makes clear that the ability to think about possibilities and act accordingly has undergone multiple critical transitions since the split of the human and chimpanzee lineages some 6 Ma. Ultimately, therefore, any striking conceptual disparities between modern humans and our closest extant primate relatives (i.e. chimpanzees) can be demystified by considering that our even closer relatives—*Ardipithecus, Paranthropus, Australopithecus, H. erectus, H. heidelbergensis,* the Neanderthals, the Denisovans, to name but a few—have all gone extinct. If even one of these hominins were still alive today, then the apparent cognitive gap between our own species and other animals would likely seem much smaller [115].

One obvious difference between modern human and non-human cognition is language. The origin of language is one of the great puzzles of evolutionary science [116,117], with many proposing that fully syntactical language emerged exclusively in *Homo sapiens* [118–120]. Whenever and however it first evolved, one likely pressure for the increasing sophistication of language was the need to communicate about actual and possible events distant from the speaker in space and time [121,122]. And in turn, language itself is likely to have driven increasing sophistication of human thinking about possibilities. As Grigoroglou & Ganea [57] highlight, linguistic and conceptual representations of possibility may feed into each other, such that language not only functions to communicate about represented possibilities, but also helps gradually transform simpler notions of possibility into more complex ones.

Vale *et al*. [69] suggest that culture and foresight similarly feed into each other, such that cultural practices assist individuals in mentally generating novel future possibilities, and in turn the individual and collective pursuit of these possibilities can transform culture. The authors therefore hypothesize that signs of advanced culture and foresight should co-occur across the animal kingdom. Nonetheless, given that culture appears so uniquely advanced in humans, it stands to reason that any bidirectional relationship between foresight and culture may likewise be uniquely potent in humans. Indeed, some of the crowning innovations of human culture are perhaps best understood as being produced by foresight and enhancing foresight in turn [123]. The invention of writing in Mesopotamia, for instance, appears to have been driven by agricultural accountants who recognized the possibility of forgetting who owed what to whom in the future [124]; subsequently, writing enabled human ideas to proliferate with high fidelity and shape the future of cultures more broadly. On the whole, it seems likely that uniquely human forms and functions of thinking about possibilities—future and otherwise—have been shaped by both natural selection and by a continual feedback loop between foresight and culture [69,123].

## 4. Conclusion

This theme issue brings together a range of scientists working on the mechanisms, ontogeny, functions and phylogeny of thinking about possibilities. It showcases cutting-edge theory and research from across several disciplines, highlighting the potential for new collaborations both within and between these disciplines. Table 1 summarizes just some of the pertinent matters arising from the issue, with many avenues for further research into proximate questions, ultimate questions and questions at the interface of proximate and ultimate perspectives. Accordingly, we trust that this compilation will provide the foundational building block for a new, integrated science of thinking about possibilities, broadly focused on understanding how this capacity works, how it develops, what it enables and how it evolved.

**Table 1.** Some critical open questions on thinking about possibilities from proximate, ultimate and interfacing perspectives.

	open questions
proximate questions: mechanisms and ontogeny	What is the relationship between the hippocampal mechanisms supporting the basic representation of possible actions in mammals, and the whole-brain networks supporting the mental construction of possible visuospatial scenarios in humans?
	Do human infants already possess a mature conceptual understanding of possibilities, or are there critical conceptual transformations during the preschool years?
	Does language play an active role in the maturation of children's conception of possibilities? If so, does this lead to conceptual differences between people from cultures with distinct linguistic markers of possibilities?
	Is there a distinction between implicit and explicit representations of possibilities, as has been proposed for representations of others' mental states?
ultimate questions: functions and phylogeny	If young children and animals tend to seek counterfactual information even without being able to reason counterfactually, then what additional benefit does counterfactual reasoning offer for learning? Is any such benefit related to the experience of counterfactual emotions like regret?
	Is counterfactual reasoning always a necessary prerequisite for causal reasoning, or are there cases where causal reasoning does not require any consideration of alternative possibilities?
	What type of archaeological findings should count as compelling evidence of thinking about possibilities in early humans? Are there different forms of evidence that would indicate thinking about single future possibilities, mutually exclusive future possibilities, and counterfactuals?
interfacing questions	Does thinking about future possibilities function to change culture, and do cultural changes in turn influence thinking about future possibilities? Can such a dynamic process be measured in an experimental setting with humans and other animals?
	Can children's emerging mechanisms for counterfactual reasoning be leveraged to enhance causal reasoning (and scientific thinking)? Does encouraging children to consider counterfactuals have long-term cognitive benefits?
	Is it possible to shift people's politically motivated counterfactual reasoning biases, in a way that might ultimately alleviate the negative social effects of heightened political polarization?

We also hope that interest in this topic will spread beyond the narrow confines of academia. Reflecting on possibilities permeates much of human life and is becoming ever more central to policymaking as our species confronts an increasingly uncertain future. Indeed, we may not live in the best of all possible worlds, as Leibniz would have it, but we can at least strive to make a better one. Achieving this goal will undoubtedly benefit from knowledge of the nature and biases of human thinking about actual and counterfactual histories, about true and imagined causal chains, and about alternative ways the future might unfold.

## Data Availability

This article has no additional data.
